# Trends in Vitamin D Status Around the World

**DOI:** 10.1002/jbm4.10585

**Published:** 2021-11-30

**Authors:** Paul Lips, Renate T. de Jongh, Natasja M. van Schoor

**Affiliations:** ^1^ Department of Internal Medicine, Endocrine Section Amsterdam University Medical Centre, location VUMC Amsterdam The Netherlands; ^2^ Department of Epidemiology and Data Science Amsterdam University Medical Centre, Vrije Universiteit Amsterdam, Amsterdam Public Health Research Institute Amsterdam The Netherlands

**Keywords:** VITAMIN D STATUS, TRENDS, 25‐HYDROXYVITAMIN D, SUPPLEMENTS, FOOD FORTIFICATION, DETERMINANTS

## Abstract

Vitamin D status varies across all continents and countries. Vitamin D status usually is adequate in Latin America and Australia, but in contrast it is very low in the Middle East and some countries in Asia. Trends in vitamin D status, whether it improves or declines over the years, carry important messages. Trends usually are small, but can be predictors and indicators of general health. Vitamin D status has improved in the older population in the United States, and improvement relates to dairy use and vitamin D supplements. To the contrary, vitamin D status has declined in the Inuit population of Canada due to a change from a traditional fish diet to a Western diet. A large improvement was seen in Finland after mandatory fortification of dairy products was introduced. Determinants of decline are less sun exposure, increased use of sunscreen, increase of body mass index (BMI), less physical activity, and poor socioeconomic status. Determinants of increase are food fortification with vitamin D and vitamin D supplements. Food fortification can lead to a population‐wide increase in vitamin D status as shown by the Finnish example. © 2021 The Authors. *JBMR Plus* published by Wiley Periodicals LLC on behalf of American Society for Bone and Mineral Research.

## Introduction

Extensive reviews on vitamin D status in the world have been published over the last decades. Although vitamin D status, as reflected by circulating 25‐hydroxyvitamin D (25(OH)D) concentrations, can be considered satisfactory in some countries, vitamin D deficiency still is very common in many countries throughout the world and in many risk groups. Guidelines have been published by the Institute of Medicine,^(^ ^1^ ^)^ the Endocrine Society,^(^ ^2^ ^)^ the European Food Safety Authority,^(^ ^3^ ^)^ and the European Calcified Tissue Society.^(^ ^4^ ^)^ What is apparent from these documents is that no consensus exists on the definition of vitamin D deficiency and the desirable serum 25(OH)D concentration. Vitamin D status appears quite stable in most countries, and percentages of vitamin D–deficient subjects have varied little during the past decades. Nevertheless, the use of supplements and the fortification of foods have generated important positive changes in some countries. On the other side, trends of increasing body mass index (BMI) and decreasing physical activity may compromise vitamin D status. In this article we give an overview of changing trends in vitamin D status in different regions and countries and the determinants that play a role in causing these changes. It is a narrative review. This article is part of a special issue of *JBMR Plus* as a tribute to Dr Anthony Norman, who made numerous contributions to the vitamin D field, was a great stimulator of laboratory and clinical investigation, and was a great mentor to many young scientists with always kind words and stimulating remarks. This article shows that both positive and negative trends in vitamin D status can occur, and these may be a reassurance or a warning for public health and policy makers.

## Methodological Issues

We searched in PubMed articles on “trends”, “temporal changes”, “vitamin D status”, “25‐hydroxyvitamin D”, “determinants” and “longitudinal studies”, covering the last 20 years. Vitamin D status is usually assessed by measuring the serum 25(OH)D concentration. This is done by radioimmunological methods, or, currently the gold standard, liquid chromatography and tandem mass spectrometry (LC‐MS/MS).^(^ ^5^ ^)^ The results of radioimmunoassays show a large variation, much more than results of LC‐MS/MS. This was demonstrated by a study using quality control data of Vitamin D External Quality Assessment Scheme (DEQAS) in which seven different methods were compared. Results for radioimmunological methods varied up to ±15% from the mean, whereas results for LC‐MS/MS varied much less, around ±5% from the mean.^(^ ^6^ ^)^ The standardization programme DEQAS has done excellent work to decrease variability,^(^ ^7^ ^)^ and more recently the Vitamin D Standardization Programme (VDSP) has brought this forward by using adequate laboratory standards.^(^ ^8^ ^)^ Longitudinal and trend studies are appropriate to compare current vitamin D status with vitamin D status one or more decades ago. Studies from different regions and time periods can be compared by remeasuring frozen samples and standardized by VDSP.^(^ ^8^ ^)^ Of course, global studies using a central laboratory facility for serum 25(OH)D can also be used to compare different countries.^(^ ^9^ ^)^ Other methodological problems arise from the assessment of sunshine exposure and vitamin D content of food.^(^ ^10^ ^)^ Vitamin D–effective ultraviolet B (UVB) availability depends on latitude, time of the day, and atmospheric conditions, whereas the actual exposure and response of the skin to this UVB in terms of vitamin D production is determined by skin pigmentation, clothing, and sunscreen use. The use of vitamin D supplements can be very effective at increasing circulating 25(OH)D, but lack of compliance decreases the effect.^(^ ^11^ ^)^ For this article, vitamin D deficiency is defined as serum 25(OH)D < 50 nmol/L, and severe vitamin D deficiency is defined as serum 25(OH)D < 30 nmol/L.

## Current Vitamin D Status

Recent reviews of worldwide vitamin D status^(^ ^12^, ^13^, ^14^ ^)^ show better vitamin D status in North and Latin America and Australia than in Europe, better vitamin D status in Southeastern Asia than in India and Northern Asia, and better vitamin D status in Central Africa than in Northern and Southern Africa. The poorest vitamin D status was generally observed in the Middle East. Within Europe, a better vitamin D status was observed in Northern Europe than in Southern and Eastern European countries.^(^ ^4^ ^)^ A global study on prevalence and disease burden of vitamin D deficiency showed high percentages of severe vitamin D deficiency in infants in India (61%), Iran (86%), and Turkey (51%), whereas vitamin D deficiency was present in 90% or higher in these countries.^(^ ^15^ ^)^

## Temporal and Regional Trends in Vitamin D Status

### North America

The National Health and Nutrition Examination Survey (NHANES) has been used to study trends. Ginde and colleagues^(^ ^16^ ^)^ compared serum 25(OH)D from NHANES collected from 1988 to 1994 with NHANES data from 2001 to 2004. A decrease of serum 25(OH)D in all age groups, both sexes and all ethnicities was observed, ranging from 7.5 to 17.5 nmol/L. Schleicher and colleagues^(^ ^17^ ^)^ repeated and extended the study by using LC‐MS/MS calibrated to a standard reference. All data were standardized and adjusted. According to this study, the serum 25(OH)D did not show a time trend from 1988 to 2006, in contrast to the previous analysis. Thereafter the mean serum 25(OH)D from 2007 to 2010 was 5–6 nmol/L higher. The largest increases (up to 11 nmol/L) were seen in older white women and in vitamin D supplement users.^(^ ^17^ ^)^ The percentage at risk for vitamin D deficiency (in this study: serum 25(OH)D < 30 nmol/L) was also estimated in NHANES in 2011–2014.^(^ ^18^ ^)^ The percentage at risk for deficiency was 0.5% in children of 1–5 years, 7.6% in adults from 20–39 years, and 2.9% in adults ≥40 years. The risk of deficiency was highest among blacks. From 2003 to 2014 there was no change in the risk of vitamin D deficiency. The risk of inadequacy (serum 25(OH)D 30–49 nmol/L) declined from 21% to 17.7%.^(^ ^18^ ^)^ Milk consumption in participants of NHANES resulted in a significantly higher serum 25(OH)D around 2–7 nmol/L.^(^ ^19^ ^)^ In the Study of Women's Health Across the Nation serum 25(OH)D was measured longitudinally in the same women in 1998–2000 and 2009–2011. Serum 25(OH)D increased from 53.8 to 70.0 nmol/L and the prevalence of vitamin D deficiency decreased from 20.4% to 9.7%.^(^ ^20^ ^)^ This was mainly due to vitamin D supplement use, which increased from 40.8% to 67.1%. A negative trend was observed in Texas. The Dallas Heart Study included 2045 participants in which serum 25(OH)D was measured in 2000–2002 and in 2007–2009. Mean serum 25(OH)D decreased from 42.7 to 39.4 nmol/L. The prevalence of vitamin D deficiency (serum 25(OH)D < 50 nmol/L) increased from 60% to 66% although vitamin D supplementation increased from 7% to 23% in that time period. Predictors of a negative change were male sex, obesity, and non‐use of vitamin D supplements.^(^ ^21^ ^)^ In general in the United States, the use of multivitamins decreased between 1999 and 2012, but the use of vitamin D supplements increased from 5.1% to 19%.^(^ ^22^ ^)^ Sun protection behavior improved in adolescents between 1998 and 2004 with possible negative effects for vitamin D status.^(^ ^23^ ^)^ An increased prevalence of rickets was observed in Minnesota during the last two decades.^(^ ^24^ ^)^

In Canada, vitamin D status was studied in the Canadian Multicenter Osteoporosis Study during 10 years starting in 1995–1997. Serum 25(OH)D increased by 9.3 nmol/L in women and by 3.5 nmol/L in men; serum 25(OH)D was lower than 50 nmol/L in 29.7% in 1995–1997 and in 19.8% 10 years later.^(^ ^25^ ^)^ A downward trend was observed in Canadian children, 6–18 years old. In 2007/2009, vitamin D deficiency (serum 25(OH)D < 50 nmol/L) was found in 21%, whereas in 2012/2013 the prevalence increased to 32%. Fish and milk consumption decreased in this time period, milk being the main dietary vitamin D source.^(^ ^26^ ^)^ Vitamin D status was assessed in the Inuit population of Greenland in 1987 and 2005–2010. Serum 25(OH)D decreased in all age groups from 32 to 58% in this period. The lowest mean serum 25(OH)D was observed in the 18–29 year group (30.7 nmol/L). Mean serum 25(OH)D increased with age. Serum 25(OH)D was lower than 50 nmol/L in 77% of 18–29‐year‐olds.^(^ ^27^ ^)^ A major determinant in this and other studies was traditional diet, consisting of fish, seal, and whale.^(^ ^28^ ^)^ The decrease in serum 25(OH)D over 20 years in the Inuit can be explained by the replacement of the traditional diet by a Western diet. Two trials with food fortification of yogurt and cheddar cheese in Canadese children showed small significant differences between intervention and control group, but mean baseline serum 25(OH)D in these children was around 60 nmol/L or higher.^(^ ^29^, ^30^ ^)^

### Latin America

Vitamin D status usually is better in Middle and South America than in North America, maybe with the exception of the more southern latitudes.^(^ ^14^ ^)^ Data on trends in vitamin D status, either decline or increase, are not available. The projected increase of the total ozone content of the atmosphere during the coming decades will lead to a decrease of the ultraviolet index at higher latitudes in South America and Antarctica, and this can have a negative impact on vitamin D status.^(^ ^31^ ^)^

### Europe

In the UK, an increase in vitamin D status between 2008 and 2016 was attributed to the prescription of supplements by primary care doctors.^(^ ^32^ ^)^ On the other side, an increase in rickets diagnosis was seen in hospital discharge data.^(^ ^33^ ^)^ The increase was restricted to children with a non‐Western immigrant background. A sharp decrease in the prevalence of vitamin D deficiency from 55% in 2014 to 14% in Irish elite athletes was observed in Ireland, also attributed to the use of supplements.^(^ ^34^ ^)^ In northern Sweden, vitamin D status was assessed in more than 11,000 men and women between 1986 and 2014. The overall mean serum 25(OH)D was 49.8 ± 23.8 nmol/L. There was no clear upward or downward trend in serum 25(OH)D concentration between 1986 and 2014.^(^ ^35^ ^)^ In Poland, vitamin D status was assessed in more than 3000 neonates and infants (mean age 8 months). The mean 25(OH)D concentration was 129.5 nmol/L between 1981 and 1999. It decreased to 107 nmol/L in 2000–2001 and to 72 nmol/L in 2010–2011.^(^ ^36^ ^)^ The decrease can be explained by a decrease of the supplementation advice from 2500 IU/d to 1000 IU/d (in breastfed children 400 IU/d). A clear improvement of vitamin D status was shown in a cohort of 1486 osteoporotic French women treated in a fracture liaison service. The mean serum 25(OH)D concentration increased from 17.6 to 48.4 nmol/L between 2005–2008 and 2009–2012 due to vitamin D supplementation.^(^ ^37^ ^)^

In the Longitudinal Aging Study Amsterdam, serum 25(OH)D was measured twice in the same participants. The mean serum 25(OH)D at baseline was 56.5 nmol/L in the younger cohort and 51.1 nmol/L in the older cohort. In the younger cohort, an increase in the mean serum 25(OH)D levels of 4 nmol/L in 6 years was observed; in the older cohort, a decrease in the mean serum 25(OH)D concentration of 4 nmol/L in 13 years was observed.^(^ ^38^ ^)^

In Germany, the results of three nationwide studies were standardized according to VDSP.^(^ ^39^ ^)^ The data of two studies in adults 18–79 years, in 1997–1999 and 2008–2011, respectively, and partially in the same subjects showed a similar percentage of severe vitamin D deficiency of 15%. However, the percentage of serum 25(OH)D between 30 and 50 nmol/L was higher in the more recent study (41%) than in the earlier study (27%), leading to the conclusion that vitamin D status has not improved, but slightly deteriorated over this time interval.^(^ ^39^ ^)^

The most interesting studies are from countries where a countrywide strategy to improve vitamin D status was used. In Finland mandatory fortification of milk and dairy products was started in 2003 with 200 IU/L of milk and yogurt and after a few years it was increased to 400 IU/L. Mean serum 25(OH)D increased from 48 nmol/L in 2000 to 65 nmol/L in 2011. In total, 91% of dairy product consumers who did not use supplements reached a serum 25(OH)D concentration >50 nmol/L. The prevalence of vitamin D deficiency in supplement non‐users decreased from 58.5 to 13.7% within a few years.^(^ ^40^ ^)^ In Turkey, a nationwide vitamin D supplementation campaign was started in 2005 to decrease the incidence of rickets in infants and toddlers. Vitamin D3 400 IU/d was given for free to all neonates. The incidence of rickets decreased from 6% to 0.1% in a few years.^(^ ^41^ ^)^

A randomized controlled trial with fortified milk in German children showed an increase of serum 25(OH)D of 8 to 15 nmol/L depending on season.^(^ ^42^ ^)^ A similar randomized controlled trial (RCT) in Swedish children of 400 and 800 IU added to 200 g of milk showed mean increases of serum 25(OH)D of 13 nmol/L and 24 nmol/L, respectively, after 3 months.^(^ ^43^ ^)^

### Middle East

The prevalence of vitamin D deficiency still is very high in the Middle East.^(^ ^4^ ^)^ A recent study from Saudi Arabia in 10,709 patients showed a prevalence of severe vitamin D deficiency (serum 25(OH)D <25 nmol/L) of 31.5%. Vitamin D deficiency was more common in women than in men. Severe deficiency was more prevalent in adolescents than in other age groups (49.2% and 30.9%, respectively). The causes are traditional clothing, low sunshine exposure, and lack of vitamin D–fortified foods.^(^ ^44^ ^)^ Vitamin D deficiency still is common in Lebanon. A database of 9147 subjects evaluated between 2000–2004 and 2007–2008 showed a prevalence of vitamin D deficiency (serum 25(OH)D <50 nmol/L) of 58%–62% in children, 44%–60% in adults, and 41%–62% in the elderly. The mean serum 25(OH)D increased 5–12 nmol/L in children and adults between the two time periods.^(^ ^45^ ^)^ A recent study of more than 150,000 serum samples standardized with cross‐calibration to LC‐MS/MS confirmed the increase of serum 25(OH)D between 2009 and 2016 of 2.2 nmol/L/year in children, 3 nmol/L/year in adults and 6.5 nmol/L/year in the elderly.^(^ ^46^ ^)^ In a longitudinal study in Iran, mean serum 25(OH)D increased from 51 nmol/L in 2001 to 54 nmol/L in 2007 and to 62 nmol/L in 2013. The prevalence of vitamin D deficiency (serum 25(OH)D <25 nmol/L) decreased from 30% to 24% in the time period of 12 years.^(^ ^47^ ^)^ The positive trend was attributed to more awareness of vitamin D deficiency, more screening, and more vitamin D supplementation.

### Africa

A recent systematic review on the prevalence of vitamin D deficiency reported a mean serum 25(OH)D of 67.8 nmol/L.^(^ ^48^ ^)^ Serum 25(OH)D was lower than 30 nmol/L in 18.5% and lower than 50 nmol/L in 34.2%. Mean serum 25(OH)D was lower in northern African countries than in sub‐Saharan African countries and South Africa. For example, mean serum 25(OH)D was 24.2 nmol/L in Algeria, 44.5 nmol/L in Morocco, and 46.5 nmol/L in Ethiopia.^(^ ^48^ ^)^ Neither increasing nor decreasing trends in vitamin D status were observed. In Morocco a double blind study with fortified milk, 3 μg (120 IU) in 200 mL, led to a sharp decrease in vitamin D deficiency from 47.6% to 11.8% in the fortified group, whereas 33% of participants in the non‐fortified group were still deficient after 9 months.^(^ ^49^ ^)^

### Asia

An analysis of 26,339 serum 25(OH)D values, obtained in a tertiary care hospital in New Delhi, India, showed an increase of the mean serum 25(OH)D from 48 nmol/L in 2008 to 54 nmol/L in 2016. The prevalence of vitamin D deficiency (serum 25(OH)D <50 nmol/L) decreased from 72% to 54% in women and from 57% to 52% in men. The improvement was attributed to greater awareness and more vitamin D supplementation.^(^ ^50^ ^)^ In some countries a negative trend in vitamin D status was observed. A study from South Korea showed a very significant decrease of vitamin D status between 2008 and 2014.^(^ ^51^ ^)^ The mean serum 25(OH)D in 2008 was 53 nmol/L in men and 46 nmol/L in women, decreasing in 2014 to 43 nmol/L in men and 39 nmol/L in women. The prevalence of vitamin D deficiency increased over the same time period. The cause of the decrease in vitamin D status is uncertain, but increased urbanization, air pollution, and less outdoor occupation were mentioned by the authors. In the Murakami Cohort Study in Japan, 1044 subjects were followed for 5 years. Serum 25(OH)D decreased 4.0 nmol/L in men and 0.4 nmol/L in women. The decline in men was related to more university education and to less outdoor occupation.^(^ ^52^ ^)^ A randomized controlled trial with fortified milk (300 IU/710 mL) in Mongolian children showed increases of serum 25(OH)D of 30 and 47 nmol/L after 1.6 months, whereas serum 25(OH)D stayed at 20 nmol/L in the control group receiving non‐fortified milk.^(^ ^53^ ^)^

### Australia, New Zealand, and Oceania

Vitamin D status is in general better in Australia and the Pacific Islands than in Asian countries.^(^ ^14^ ^)^ Vitamin D status is less in New Zealand than in Australia due to the more southern latitude.

Persons who tended to stay in the shade had lower serum 25(OH)D levels than those who never stayed in the shade (62.5 versus 68.8 nmol/L, respectively, *p* = 0.01), and this association remained in persons who spent <50% (*p* = 0.02), but not in those who spent ≥50% of their time outdoors.^(^ ^54^ ^)^ Trends in sun protection behavior were not observed in a study between 2007 and 2012, except an increase in sunscreen use.^(^ ^55^ ^)^ In another study, a decline in skin covering around swimming pools and beaches was observed between 2006 and 2019.^(^ ^56^ ^)^

## Determinants of Change in Vitamin D Status

**Fig. 1:**
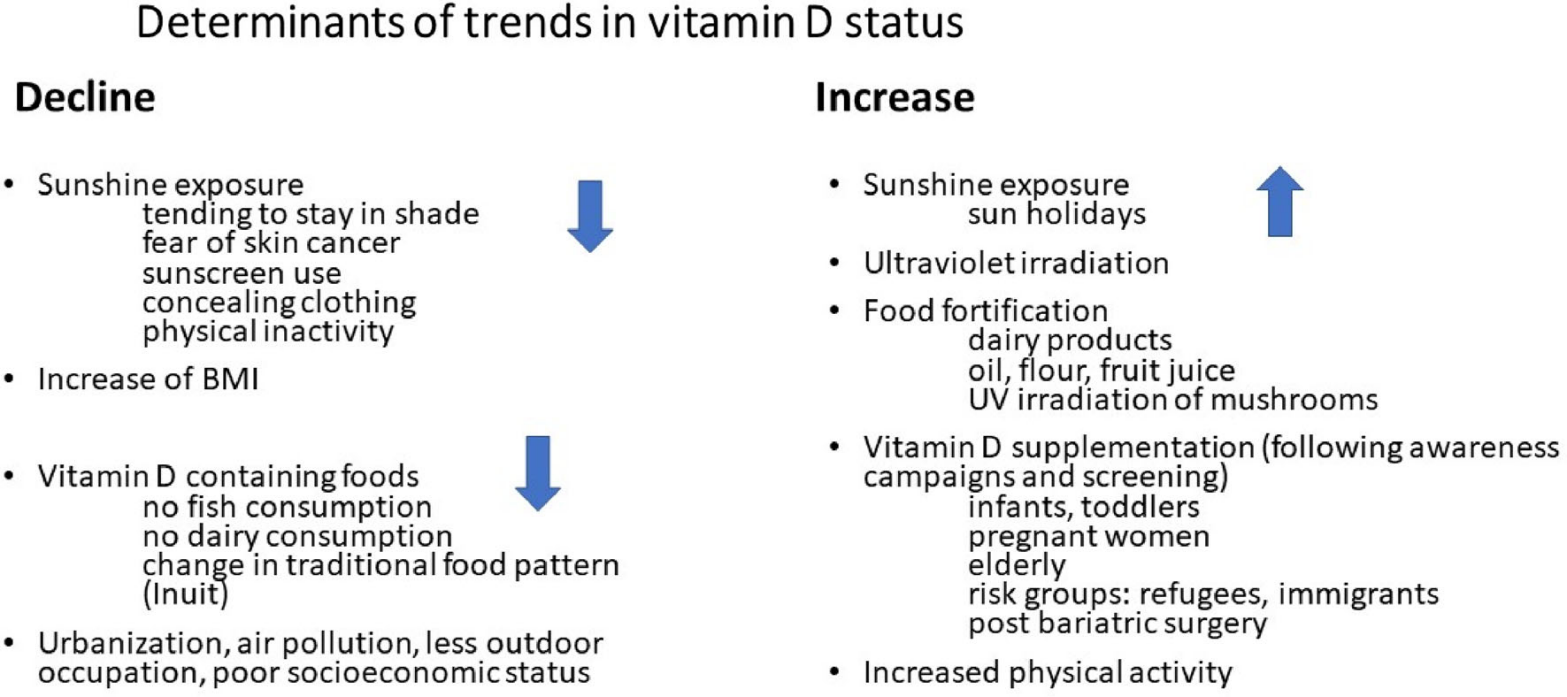
Determinants of decline or increase in vitamin D status.

Trends in vitamin D status, either decline or increase, are determined by many factors (Fig. 1). A negative trend of vitamin D status with aging was seen in the Longitudinal Aging Study Amsterdam (LASA).^(^ ^38^ ^)^ Of course, vitamin D deficiency is very common in the elderly.^(^ ^57^ ^)^ Little data exists on trends in sun exposure. Sun protection behavior increases somewhat according to American and Australian studies.^(^ ^23^, ^55^ ^)^ However, the Australian data are ambiguous.^(^ ^56^ ^)^ The use of sunscreen may increase, leading to lower vitamin D production.^(^ ^58^ ^)^ Clothing style is an important determinant of vitamin D status according to studies in the Middle East, Jordan,^(^ ^59^ ^)^ and Turkey.^(^ ^60^ ^)^ Total skin covering clothes may also explain the very poor vitamin D status in Saudi Arabia.^(^ ^61^, ^62^ ^)^ Urbanization also may decrease vitamin D status. Vitamin D status usually is better in rural than in urban areas as observed in Mexico,^(^ ^63^ ^)^ Malaysia,^(^ ^64^ ^)^ South Africa,^(^ ^65^ ^)^ and Korea.^(^ ^66^ ^)^ As vitamin D status is less in obese people,^(^ ^67^ ^)^ increasing obesity may deteriorate vitamin D status. A very large multicenter survey found a trend for increase of BMI in children and adolescents.^(^ ^68^ ^)^ Contrary to expectation, BMI rose more in rural than in urban areas.^(^ ^69^ ^)^ This effect counteracts the higher vitamin D status in rural versus urban areas. In LASA, subsequent cohorts from 1992/1993 to 2002/2003 to 2012/2013 of participants of 55–64 years of age showed that unhealthy lifestyle increased, visible by increasing BMI and decreasing physical activity.^(^ ^70^ ^)^ Both factors may contribute to a decline in vitamin D status. In Japan, decline in vitamin D status was less in men with an outdoor occupation and the highest physical activity.^(^ ^52^ ^)^ Serum 25(OH)D also relates positively to socioeconomic status as measured by a housing score, Houses Index.^(^ ^71^ ^)^

Nutritional changes may also cause a decline in vitamin D status. The Inuit in Canada changed from a traditional to a Western diet and vitamin D status deteriorated. The traditional Inuit diet contains fish and sea mammals, both rich in vitamin D.^(^ ^28^ ^)^ The effects of the mandatory fortification of dairy products with vitamin D was particularly well documented in Finland, where mean serum 25(OH)D increased with 17 nmol/L.^(^ ^40^ ^)^ The voluntary fortification of dairy products in the United States may explain the better vitamin D status in the United States than in Europe. Randomized controlled trials with fortified milk, yogurt or cheese were highly successful.^(^ ^72^ ^)^ Vitamin D–fortified orange juice was studied in the United States and increased serum 25(OH)D about 25 nmol/L.^(^ ^73^ ^)^ It now is available in many countries. Fortification of flour and cooking oil is practiced in the Middle East,^(^ ^74^, ^75^ ^)^ and fortification of milk, oil, and rice with vitamin D is now practiced in India.^(^ ^76^ ^)^ Modeling of fortification policies lead to the conclusion that several methods can be successful.^(^ ^77^ ^)^ Of course, the use of vitamin D supplements can also increase vitamin D status (see temporal and regional trends in vitamin D status). A very successful supplementation campaign was done among neonates in Turkey to eradicate rickets.^(^ ^41^ ^)^

## Conclusions

Trends in vitamin D status, either decline or increase, can be observed in several countries under various circumstances. Usually these trends are small, but they can be important predictors for future development and indicators of general health change. Small improvements of vitamin D status as observed in the United States can be caused by vitamin D supplement use. A large improvement of vitamin D status as observed in Finland is due to fortification of dairy products with vitamin D. A decline in vitamin D status can be caused by nutritional changes as is the case in the Inuit population in Canada. In general, a decrease in vitamin D status can be due to an increase in BMI and decrease in physical activity, usually associated with less time spent outdoors. Such a trend is a warning for health authorities and policy makers.

## Conflict of Interest

PL reports a travel grant from Abiogen. RJ and NS do not have a conflict of interest.

### Peer Review

The peer review history for this article is available at https://publons.com/publon/10.1002/jbm4.10585.
